# Editorial: Highlights in heart valve disease: 2021

**DOI:** 10.3389/fcvm.2022.955596

**Published:** 2022-08-12

**Authors:** Aikawa Elena

**Affiliations:** Cardiovascular Medicine, Brigham and Women's Hospital, Harvard Medical School, Boston, MA, United States

**Keywords:** rheumatic heart valve disease, antithrombocytic therapy, transcather aortic valve replacement, aortic valve stenosis, mitral valve disease

This collection brings together a selection of the top articles published in 2021 in the Heart Valve Disease Section of Frontiers of Cardiovascular Medicine (Table 1).

**Table 1:** Top articles published in 2021 at Heart Valve Disease Section.

**Category**	**Author**	**Number of views (June 2022)**	**Number of citations (June 2022)**
Infection	Yamamoto et al.	2,245	1
	Vinciguerra et al.	3,220	3
Anticoagulation therapy	Myllykangas et al.	2,009	1
	Verstraete et al.	7,895	3
TAVR	Tamm et al.	2,101	4

The number of views and citations is provided for up to when this editorial was finalized.

The authors' names are listed in order of appearance in the text.

Despite the wide use of antibiotics in the industrialized world, *Streptococci* remains the main cause of rheumatic heart valve disease. In the contribution by Yamamoto et al., a case report describing a miniature erupting volcano-shaped mitral valve aneurysm secondary to *Streptococcus Agalactiae* ST1656 endocarditis is discussed. Mitral valve aneurysm is a rare life-threatening condition causing an aneurysmal rupture and systemic embolization and is commonly associated with infective endocarditis and involvement of the aortic valve. In this report, a diabetic patient with a ruptured mitral valve aneurysm presented serious complications requiring early surgical intervention instructed by transesophageal echocardiography. The patient underwent mitral valve replacement with a prosthetic valve. The aortic valve was not involved. Overall, this case report underscores the importance of early treatment of mitral valve aneurysm as a potential consequence of infectious endocarditis in elderly patients with comorbidities and raises awareness for early diagnosis of this disease.

On the other hand, a case report by Vinciguerra et al., suggests that another group of *Streptococci*- *Streptococcus Alactolyticus* is an uncommon pathogen in mitral valve disease. This pathogen affects distant organs and is extremely rare and has been described as causing infective endocarditis in the mitral valve only in two other cases. The strong association between *Streptococcus Alactolyticus and* infection in gastrointestinal disease facilitated by surgery could allow the translocation of the pathogen toward the target organs comprising of collagen-rich tissue such as heart valves. This case emphasizes the importance of appropriate imaging techniques and early diagnosis in the assessment of disease progression to optimize the treatment of infective endocarditis.

Calcific aortic valve stenosis is a major and growing health burden in the developed world, and valve intervention is the only effective treatment. Currently, approximately 300,000 artificial valves are implanted every year, and it is estimated that this number will increase further. Artificial valves require antithrombotic treatment with warfarin or low-dose aspirin to prevent thromboembolism. The peak incidence of thromboembolic complications occurs within 3 months postoperatively, likely due to the lack of formation of new endothelium on the surface of the newly implanted materials. However, anticoagulation therapy often continues longer to prevent stroke and other thromboembolic complications. In this article, Myllykangas et al. examined the continuation of oral anticoagulation treatment and its effect on long-term prognosis after aortic valve replacement (AVR). The study used nationwide register data obtained between 2010 and 2016 from 4,079 AVR patients. The association between warfarin and the non-vitamin K antagonist oral anticoagulant use and death, stroke, and major bleeding was examined. The use of oral anticoagulants after AVR surgeries is associated with an increased risk of stroke and decreased risk of death. There was no association between anticoagulant use and bleeding risk. The validation of these findings in randomized controlled trials has been recommended.

The following article by Verstraete et al. emphasizes that decision-making regarding anticoagulation therapy for prosthetic heart valves is challenging due to contradictory recommendations in current guidelines and opposing expert opinions. The article compares 2017 ESC/EACTS and 2020 ACC/AHA guidelines on the management of heart valve disease. The authors established a consensus on antithrombotic therapy after valve interventions based on 800 cases of surgical and transcatheter heart valve replacement (TAVR), which provides practical guidance for decision-making in daily clinical practice at University Hospital Leuven, Belgium. The article also provides a list of Unsolved Questions and Starting Points for Research. As such, this article offers standard guidelines and delivers clear recommendations for antithrombotic treatment after surgical or transcatheter heart valve interventions from 2021 onwards, which will be updated once new data become available.

TAVR has evolved into standard treatment for patients with severe aortic valve stenosis. Paravalvular regurgitation, however, has been associated with a worth survival. A team from University Mainz, Germany led by Tamm et al. investigated the performance and outcome of the latest generation balloon-expandable SAPIEN 3 Ultra prosthesis (S3U) compared to established SAPIEN 3 device (S3) in a real-world cohort. The study focused on paravalvular regurgitation as an adverse prognostic indicator for survival after TAVR. Three hundred forty-three patients with severe aortic valve stenosis received either S3U or S3 devices. The authors found that the novel S3U prostheses significantly minimized paravalvular regurgitation indicating an improved prosthesis design. In addition, superior performance was reported for post-dilation rate, radiation time, and the amount of contrast as well as relief of symptoms at 30 days. Future studies will further illuminate its prognostic significance.

These articles identified several critical areas of clinical and surgical research interests, including the importance of early diagnosis of mitral valve disease in patients with infectious endocarditis, the inconsistency in management of anticoagulation therapy after surgical valve replacement and TAVR, and the necessity of innovative approaches to improve novel devices to treat heart valve disease.

## Author contributions

The author confirms being the sole contributor of this work and has approved it for publication.

## Funding

This work was supported by National Institutes of Health grants (NIH) R01HL147095, R01HL141917, and R01HL136431 to AE.

## Conflict of interest

The author declares that the research was conducted in the absence of any commercial or financial relationships that could be construed as a potential conflict of interest.

## Publisher's note

All claims expressed in this article are solely those of the authors and do not necessarily represent those of their affiliated organizations, or those of the publisher, the editors and the reviewers. Any product that may be evaluated in this article, or claim that may be made by its manufacturer, is not guaranteed or endorsed by the publisher.

